# *Aspergillus* PCR in Bronchoalveolar Lavage Fluid for the Diagnosis and Prognosis of Aspergillosis in Patients With Hematological and Non-hematological Conditions

**DOI:** 10.3389/fmicb.2018.01877

**Published:** 2018-08-14

**Authors:** Sébastien Imbert, Isabelle Meyer, Martine Palous, Jean-Yves Brossas, Madalina Uzunov, Feriel Touafek, Frédérick Gay, Valéry Trosini-Desert, Arnaud Fekkar

**Affiliations:** ^1^AP-HP, Groupe Hospitalier Pitié-Salpêtrière, Service de Parasitologie Mycologie, Paris, France; ^2^Centre d’Immunologie et des Maladies Infectieuses, Paris, France; ^3^AP-HP, Hôpital Pitié-Salpêtrière, Sorbonne Université, Paris, France; ^4^AP-HP, Groupe Hospitalier Pitié-Salpêtrière, Service d’Hématologie, Paris, France; ^5^AP-HP, Groupe Hospitalier Pitié-Salpêtrière, Service de Pneumologie, Paris, France

**Keywords:** *Aspergillus fumigatus*, PCR, fungal infection, solid organ transplant, hematology, galactomannan

## Abstract

**Objectives:** We evaluated the usefulness of an *Aspergillus fumigatus* quantitative PCR assay performed in bronchoalveolar lavage fluid (BAL) for the diagnosis and prognosis of both invasive and non-invasive aspergillosis.

**Methods:** This 4-year retrospective study involved 613 at-risk patients who had either hematological disorders or other immunosuppressive conditions, notably solid organ transplants. Thirty-five patients had proven/probable aspergillosis and thirteen had chronic non-invasive aspergillosis. We compared PCR, galactomannan index and mycological analysis of BAL.

**Results:** For invasive aspergillosis (IA), PCR performed in BAL yielded 88.6% sensitivity and 95.5% specificity. Comparatively, galactomannan index and mycological examination yielded only 56.3 and 63.6% sensitivity and 97.6 and 94.5% specificity, respectively. Considering the 13 chronic aspergillosis cases, PCR, galactomannan index and mycological examination yielded 76.9, 15.4, and 84.6% sensitivity and 92.2, 94.9, and 93% specificity, respectively. Fungal load in BAL evaluated by PCR was able to discriminate between aspergillosis and contamination, but not between invasive and non-invasive forms. Finally, fungal load was predictive of 90-day mortality, with 23.1% mortality for patients with less than 500 copies/mL versus 68.4% for patients above that cut-off (*p* < 0.05).

**Conclusion:** Our results indicate that *Aspergillus* PCR in BAL is of particular interest for both the diagnosis and the prognosis of IA. It is likewise an interesting tool for the diagnosis of non-invasive forms.

## Introduction

Invasive aspergillosis (IA) is a particularly severe disease in immunocompromised patients. Its diagnosis remains difficult and is often based on a body of arguments built around host, clinical, radiological and mycological criteria such as those defined jointly by the European Organisation for Research and Treatment of Cancer and the National Institute of Allergy and Infectious Diseases Mycoses Study Group (EORTC/MSG) (De Pauw et al., 2008). Although not included in these criteria, the detection of *Aspergillus* DNA in blood samples by real-time polymerase chain reaction (PCR) has a long history of use and has been shown to be a very interesting test for diagnosing IA in both neutropenic and non-neutropenic patients (Suarez et al., 2008; Imbert et al., 2016). Moreover, this approach has been shown to have predictive ability for disease outcome (Imbert et al., 2016). There is now a consensus among experts for its inclusion in the EORTC/MSG criteria (White et al., 2015; Patterson et al., 2016; Ullmann et al., 2018). Even if less sampled because of their invasiveness, respiratory samples, such as bronchoalveolar lavage (BAL) fluid, may be useful for IA diagnosis. EORTC/MSG recommend the use of galactomannan in BAL as a mycological criterion (De Pauw et al., 2008), even if studies show heterogeneity in performances and the optimal cut-off value is not definitively determined (Zou et al., 2012). *Aspergillus* PCR is also practicable in BAL samples. Some studies showed promising performances, however, less is known concerning its potential usefulness (Tuon, 2007; Zou et al., 2012; Grancini et al., 2018; Guegan et al., 2018).

Otherwise, non-invasive aspergillosis can occur in non-immunocompromised patients and comprises several clinical forms with variable severity, including chronic pulmonary aspergillosis (CPA) and allergic diseases as allergic broncho-pulmonary aspergillosis (ABPA) (Kosmidis and Denning, 2015). As IA, their diagnosis is difficult and relies mainly on clinical and radiological findings, associated with an evidence of *Aspergillus* in respiratory samples or with a positivity of anti-*Aspergillus* antibody in serum. Guidelines edited jointly by the European Society of Clinical Microbiology and Infectious Diseases and the European Respiratory Society (ESCMID/ERS) recommend the use of *Aspergillus* PCR in BAL for the diagnosis of non-invasive aspergillosis with C-II grade (Denning et al., 2016).

Thus, for the present study, we evaluated an in-house *A. fumigatus* real-time PCR assay, in comparison to the galactomannan (GM) assay and mycological examination, for the diagnosis of both invasive and non-invasive forms of aspergillosis in at-risk patients. We also assessed the contribution of PCR to the 90-day prognosis.

## Materials and Methods

### Design

A 4-year, retrospective, single-center analysis was performed between February 2012 and February 2016 in La Pitié-Salpêtrière hospital, a tertiary care center in Paris, France.

### Patients

All patients in whom at least an *Aspergillus* PCR was performed on BAL due to a risk of IA or a suspicion of a non-invasive form of aspergillosis were included in the study. Results of BAL mycological analysis (direct examination and culture), GM (serum and BAL) and PCR (serum and BAL) were obtained from routine clinical practice in accordance to clinician’s prescription at the sample time. No analyses were performed on stored sample. Clinical and radiological data were collected retrospectively. We thereafter excluded from analyses patients for whom data were lacking. The study analyzes results were obtained from routine clinical practice, so no specific authorization from a research ethics committee is required.

The present study was focused on patients with proven/probable IA according to the extended EORTC/MSG criteria (De Pauw et al., 2008) or with non-invasive aspergillosis. Possible IA cases were excluded from the study. A BAL positive culture for *Aspergillus* sp., a positive GM index in serum (index > 0.5), and a positive PCR in serum were used as mycological criteria for probable cases, and risk factors now known to lead to invasive aspergillosis (liver cirrhosis, severe acute respiratory distress syndrome, extracorporeal membrane oxygenation, terminal chronic obstructive pulmonary disease and terminal solid malignancy), were added to the host factors. To avoid inclusion bias, GM and PCR results on BAL were excluded from mycological criteria. Diagnosis of non-invasive forms was made in patients for whom compatible clinical and radiological findings were present in association with a positive *Aspergillus* culture in BAL and the presence of seric anti-*Aspergillus* antibody in serum.

#### PCR

The assessed real-time PCR assay targets a 67-bp segment of a 28S ribosomal RNA coding DNA and was used as previously described (Challier et al., 2004; Suarez et al., 2008; Imbert et al., 2016). DNA extraction was performed on 1 mL of BAL with the MagNA Pure Compact large volume kit on a MagNA Pure device (Roche). Elution volume was 50 μL. Amplification was performed on the 7500 Fast Real-Time PCR System (Applied Biosystems). Quantification was achieved using five serial 10-fold dilutions of the plasmid PGEMT Easy-Afu28S containing the target. The final PCR result was expressed in numbers of copies per mL of sample. An internal control was used in the assay for all wells (TaqMan exogenous Internal Positive Control) as an extraction control (albumin gene) for each sample. All PCRs were performed in duplicate. A single positive well was considered a positive result.

### Galactomannan Determination

The GM index was determined by enzyme immunoassay (BioRad) according to the manufacturer’s recommendations. A result was considered positive after two determinations, performed on two different assays but on the same sample, showing both an index equal to or greater than 0.5 for serum and an index equal to or greater than 1 for BAL.

### Statistical Analysis

Tests were performed using GraphPad Prism 5 and the free online site BiostaTGV^1^. Categorical variables were compared using the Chi^2^ test or Fischer’s exact test. Fungal loads were compared using the Student’s *t*-test and survival distribution using the Log-Rank test. A *p*-value less than 0.05 was considered to be statistically significant.

## Results

### Patient Characteristics

**FIGURE 1:**
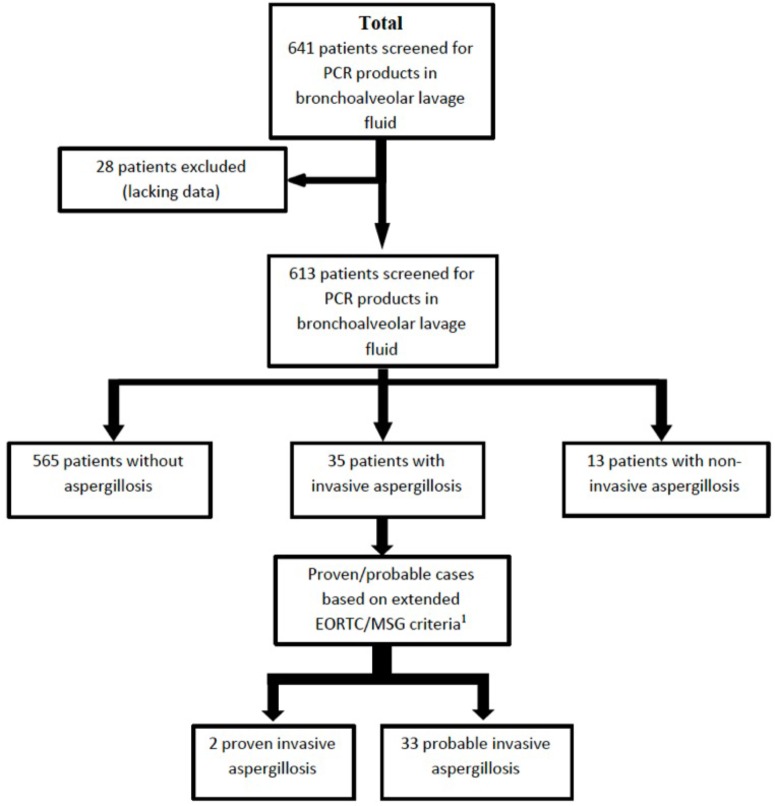
Flow chart illustrating the number of patients and samples included in the study. ^1^Extended EORTC/MSG criteria included host factors as published in 2008 plus several other host factors now recognized as leading to a risk of developing invasive aspergillosis, namely alcoholic liver cirrhosis, severe acute respiratory syndrome, long stay in intensive care unit, and solid organ cancer.

Over the study period, *A. fumigatus* PCR was performed in 641 patients (**Figure 1**). Clinical data were available for 613 patients (785 BAL samples). Diagnoses of proven or probable IA were made respectively for 2 and 33 patients according to the extended EORTC/MSG criteria including serum PCR as a mycological criterion, which was the only mycological criterion for 4 patients only. Two of the probable cases did not involve the lung: one was a digestive aspergillosis with many culture-positive samples and the other of unknown origin with positive GM and PCR results in serum and a normal full thoracic CT-scan. A non-invasive form of aspergillosis was diagnosed in 13 patients. The overall, invasive and chronic aspergillosis incidences were respectively 7.8, 5.7, and 2.1% over the study period.

### Characteristics and Outcomes of Patients With Invasive and Non-invasive Aspergillosis

Among the 35 patients with proven/probable invasive aspergillosis, there were 12 females and 23 males (**Table 1**). Their median age was 59 years (range 23–74). Sixteen patients (45.7%) received corticoid therapy and ten patients (28.6%) were neutropenic (absolute neutrophil count < 500/μL) at the time of diagnosis. Underlying conditions were mainly solid organ transplantation (*n* = 12; 34.2%: heart = 6, liver = 2, kidney = 2, liver/kidney = 1), haematopoietic stem cell transplantation (*n* = 6; 17.1%) and hematological malignancies (*n* = 6; 17.1%). Others risk factors were present in 11 patients: severe acute respiratory distress syndrome with ECMO (*n* = 4; 11.4%), oncological diseases (*n* = 3; 8.6%), alcoholic liver cirrhosis (*n* = 2; 5.7%), cardiogenic shock with ECMO (*n* = 1; 2.9%), terminal chronic obstructive bronchopneumonia (*n* = 1; 2.9%). The overall 3-month mortality of invasive aspergillosis was 54.3% (19/35).

**Table 1:** Characteristics of patients with proven/probable invasive aspergillosis according to extended EORTC/MSG criteria and with non-invasive form of aspergillosis.

	Invasive aspergillosis				Non-invasive aspergillosis
	All (*n* = 35)	*A. fumigatus* IPA (*n* = 32)			
		<500 copies/ml	≥500 copies/ml	*p*	
Patients (n)	35	13	19		13
Median age (years)	59	57	54	0.8	63
Sex ratio (Male/Female)	23/12	10/3	10/9	0.27	10/3
**Underlying disease**					
Hematological malignancy (%)	6 (17.1)	2 (15.4)	3 (15.8)	1	0
HSCT (%)	6 (17.1)	2 (15.4)	4 (21.1)	1	0
SOT (%)	12 (34.3)	6 (46.2)	5 (26.3)	0.28	4 (30.8)
Solid malignancy (%)	3 (8.6)	1 (7.7)	1 (5.3)	0.47	1 (7.7)
Terminal cirrhosis (%)	2 (5.7)	0	2 (10.5)		0
Bronchopulmonary disease (%)	1 (2.9)	0	1 (5.3)		8 (61.5)
ICU ECMO (%)	5 (14.3)	2 (15.4)	3 (15.8)		0
Neutropenia (absolute neutrophil count < 500/μL)	10 (28.6)	4 (30.8)	5 (26.3)	1	0
Corticosteroid therapy (>0.3 mg/kg/d)	16 (45.7)	5 (38.5)	9 (47.4)	0.72	5 (38.5)
Azole treatment (after diagnosis)	30 (85.7)	12 (92.3)	15 (78.9)	0.62	10 (76.9)
Interval between time of sample and start of antifungal therapy (days)	2.29	3.25	1.78	0.6	
30-day mortality (%)	15 (42.9)	2 (15.4)	10 (52.6)	0.06	0
3-month mortality (%)	19 (54.3)	3 (23.1)	13 (68.4)	0.03	1 (7.7)

*Characteristics of patients with invasive pulmonary aspergillosis according to the *Aspergillus* PCR level are also shown.*

Among the 13 patients with non-invasive aspergillosis, clinical forms were CPA for 9 patients (69.2%), including chronic cavitary pulmonary aspergillosis (*n* = 5; 38.5%), aspergilloma (*n* = 3; 23.1%) and chronic necrotising pulmonary aspergillosis (*n* = 1; 7.7%). One patient (7.7%) had a sinus aspergilloma, 2 patients (15.7%) an aspergillus bronchitis and the last one (7.7%) an allergic bronchopulmonary aspergillosis. Three-month mortality was 7.7% (1/13), a rate significantly lower than that of invasive forms (*p* < 0.01 by Fisher’s exact test).

### Performance of the Galactomannan Index, Mycological Examination and PCR on BAL for the Diagnosis of Invasive Aspergillosis

**FIGURE 2:**
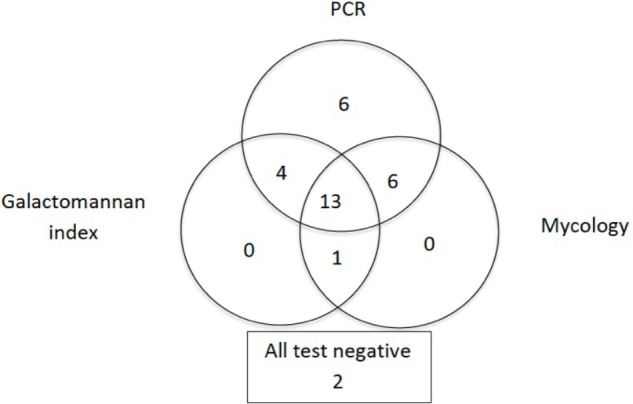
Venn diagram showing data for patients with invasive aspergillosis (*n* = 32). Diagram shows data for patients for whom PCR products, galactomannan and mycological analysis of bronchoalveolar lavage fluid were available on a same sample. For two patients, all tests were negative.

Proven/probable invasive aspergillosis (according to the extended EORTC/MSG criteria) was diagnosed in 35 patients. PCR was positive in 31 of these 35 patients. However, the three methods were performed on a same BAL sample for 32 patients (**Figure 2**). Sensitivities for PCR, the GM index and mycological examination were 88.6% (95% confidence interval [CI], 78.1–99.1%), 56.3% (95% CI, 39.1–73.5%), and 63.6% (95% CI, 47.2–80.0%) respectively (**Table 2**). Therefore, PCR provided significantly better sensitivity than the GM index (*p* < 0.005) or mycological examination (*p* = 0.01). Of note, GM in serum was positive (index ≥ 0.5) in 19/31 of these IA patients (sensitivity of 61%).

**Table 2:** Performance of *Aspergillus fumigatus* PCR, galactomannan index determination and mycological examination of respiratory samples for the diagnosis of aspergillosis in 35 patients with proven/probable invasive aspergillosis according to extended EORTC/MSG criteria and in 13 patients with non-invasive chronic pulmonary aspergillosis.

Clinical form	Method	Number of samples with result		Sensitivity (%)	*p*-value^a^	Specificity (%)	*p*-value^a^	Positive predictive value (%)	Negative predictive value (%)
		Positive	Negative						
All aspergillosis	PCR	41	7	85.4		97.2		71.9	98.7
	GM	20	25	44.4	<0.001	97.9	0.41	64.5	95.4
	Mycology (direct examination) ^b^	32 (15)	14 (31)	69.6	0.06	96.4	0.45	61.5	97.4
Invasive aspergillosis	PCR	31	4	88.6		95.5		54.4	99.3
	GM	18	14	56.3	0.003	97.6	0.05	58.1	97.4
	Mycology (direct examination)	21 (11)	12 (22)	63.6	0.01	94.5	0.44	40.4	97.8
Chronic pulmonary aspergillosis	PCR	10	3	76.9		92.2		17.5	99.5
	GM	2	11	15.4	0.002	94.9	0.06	6.5	98.0
	Mycology (direct examination)	11 (4)	2 (9)	84.6	0.62	93.0	0.59	21.2	99.6
No aspergillosis	PCR	16	549	–	–	–	–	–	–
	GM	11	523	–	–	–	–	–	–
	Mycology (direct examination)	20 (0)	532 (0)	–	–	–	–	–	–

*^a^p-value for comparison with PCR results, calculated by Chi-square test. ^b^Mycological examination (‘mycology’ in the table) included microscopic analysis and a 28-day Sabouraud agar culture of respiratory samples.*

Excluding the 4 patients in whom serum PCR was the only mycological criterion, lead to the same results with sensitivity for BAL PCR, BAL GM index and BAL mycological examination of 87% (95% CI, 75.2–98.8%), 62% (95% CI, 44–80%) and 70% (95% CI, 53–87%), respectively. Specificities for PCR, the GM index and mycology examination were 95.5% (95% CI, 93.8–97.2%), 97.6% (95% CI, 96.3–98.9%) and 94.5% (95% CI, 92.6–96.4%), respectively. Of note, decreasing the GM index positivity cut-off led to an increased sensitivity (68.7% [95% CI, 52.6–84.8%]), but still lower than PCR (*p* = 0.04), and a decreased specificity (93.8% [95% CI, 91.8–95.8%]). However, more than the half of the BAL GM index comprised between 0.5 and 1 were false positive (20/24). One of the four PCR-negative invasive cases was caused by *A.* *nidulans*, a species not detected by the PCR used in this study, and the two others showed no pulmonary involvement. Therefore, the sensitivity of the PCR for the pulmonary form of *A. fumigatus* IA was 96.9% (31/32) in this series.

Adding either mycological examination or GM determination to PCR slightly improved sensitivity (especially for non-*A. fumigatus* IA), but combining the three methods did not. PCR was the only positive test in BAL for 6 IA patients, whereas it was never the case for GM and mycological examination. Therefore, excluding PCR from the combination led to a decreased sensitivity (**Figure 2**).

### Neutropenic Versus Non-neutropenic

No statistically significant differences were observed between neutropenic and non-neutropenic patients in the group of 35 patients with proven/probable IA. Sensitivities for PCR, the GM index and mycological examination were 90% (*n* = 9/10), 50% (*n* = 5/10) and 40% (4/10) in neutropenic patients and 88% (*n* = 22/25), 52% (*n* = 13/25) and 68% (17/25) in non-neutropenic patients, respectively (data not shown). Their specificities were 94.1% (*n* = 16/17), 100% (*n* = 17/17) and 100% (17/17) in neutropenic patients and 95.5% (*n* = 536/561), 97.5% (*n* = 514/527) and 94.3% (515/546) in non-neutropenic patients, respectively.

### PCR Results for Non-invasive Forms of Aspergillosis

PCR, the GM index and mycological examination were positive in respectively 10, 2 and 11 of the 13 cases of non-invasive chronic aspergillosis. Therefore, the sensitivity of PCR (76.9% CI, 54–99.8%) was considerably superior to that of the GM index (15.4% CI, 0–35%) (*p* < 0.005) and similar to that of mycological examination (84.6% CI, 65–100%). Positive predictive values (PPV) for all forms of aspergillosis were 71.9% (95% CI, 60.2–83.6%), 64.5% (95% CI, 47.7–81.3%) and 61.5% (95% CI, 48.3–74.7%) for PCR, GM and mycological examination respectively. However, considering these tools for the diagnosis of IA only, leads to decreased PPVs (**Table 2**). Indeed, for the invasive forms, PCR yielded 54.4% PPV (95% CI, 41.5–67.3%) and 99.3% negative predictive value (NPV) (95% CI, 98.6–100%), while the GM index yielded 58.1% PPV (95% CI, 40.7–75.5%) and 97.4% NPV (95% CI, 96.1–98.7%) and mycological examination 40.4% PPV (95% CI, 27.1–53.7%) and 97.8% NPV (95% CI, 96.6–99.0%). It is noteworthy that the three methods provided a high NPV (between 95.4 and 99.6%), whatever the clinical form of aspergillosis.

### Effect of Antifungal Therapy

Among the 35 patients with IA, 17 patients were free from antifungal therapy at the time the BAL was sampled, while the 18 others patients were receiving an antifungal treatment: 10 patients received voriconazole, 4 received liposomal amphotericin B, 2 received voriconazole plus liposomal amphotericin B, 1 patient had caspofungin and 1 patient was given posaconazole. Average duration of mold-active drug before sampling was 7.5 days (1–28 days). GM values in BAL were available for 32 patients and show no difference between the non-treated group and the patients who received antifungal drug before BAL sampling (mean value 1.9 versus 1.8, respectively; *p* = 0.9 by Mann Whitney test). PCR sensitivity in BAL was not different between the groups: 88.2% (15/17) for non-treated group and 88.9% (16/18) for patients under antifungal treatment (*p* = 1). Similarly, GM sensitivity in BAL was not affected: 53.3% (8/15) among patients without antifungal therapy and 58.8% (10/17) for patients receiving antifungal drug before sampling (*p* = 1). The only parameter affected between patients with and without a previous mold-active antifungal therapy, was the BAL culture, with a sensitivity of 47% (8/17) and 81.3% (13/16) respectively. The difference was statistically significant by Chi-squared test (*p* = 0.04).

### Assessment of the Fungal Load for Discrimination Between Aspergillosis and Non-aspergillosis Pathologies

**FIGURE 3:**
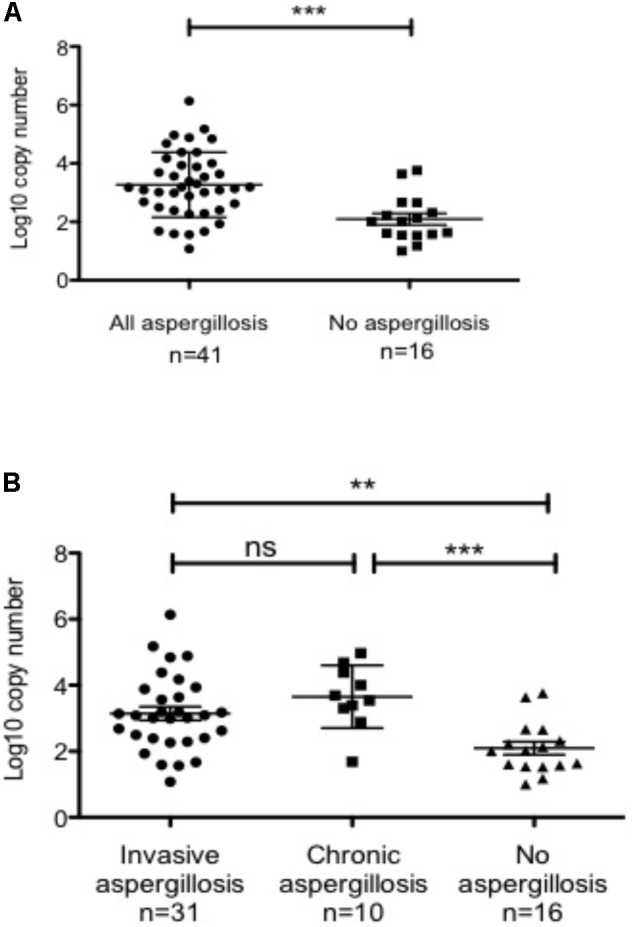
Fungal load assessed by *Aspergillus* PCR in bronchoalveolar lavage fluid can discriminate between aspergillosis and contamination/clinically irrelevant colonization. **(A)** Patients with aspergillosis had higher *Aspergillus* PCR copy numbers compared to patients for whom aspergillosis was excluded; ^∗∗∗^*p* < 0.0005 by *t*-test. **(B)** Patients with either invasive or chronic forms of aspergillosis had higher *Aspergillus* copy numbers compared to patients for whom aspergillosis was excluded. However, no significant differences were identified between patients with invasive or chronic forms of aspergillosis. ^∗∗^*p* < 0.005 by *t*-test; ^∗∗∗^*p* < 0.0005 by *t*-test. Error bars represent mean and standard deviation.

PCR is a very sensitive method and can be positive in non-aspergillosis conditions, due either to the contamination of the sample by airborne conidia or the presence of fortuitous and non-specifically pathogenic *Aspergillus* in the airway. Moreover, real-time PCR can quantify fungal load, with results expressed as the number of *Aspergillus* gene copies per mL of BAL. Considering this, we assessed the ability of the fungal load to predict the clinical form of aspergillosis and to distinguish between invasive, non-invasive and non-aspergillosis diseases. The analysis of PCR-positive BAL and numbers of copies expressed in Log10 showed that this latter was significantly higher in BAL sampled from patients with aspergillosis, in comparison with BAL sampled from patients with non-aspergillosis conditions (*p* < 0.0005 by *t*-test; **Figure 3A**). However, the numbers of copies were not statistically different (*p* = 0.2 by *t*-test) between invasive and chronic forms, although each of them was related to a statistically significantly higher fungal load in comparison with the non-aspergillosis PCR-positive group (**Figure 3B**). These results suggest that fungal load might be useful to discriminate between aspergillosis and contamination/clinically irrelevant colonization.

### BAL Fungal Load and Prediction of Outcome of Invasive Pulmonary Aspergillosis

**FIGURE 4:**
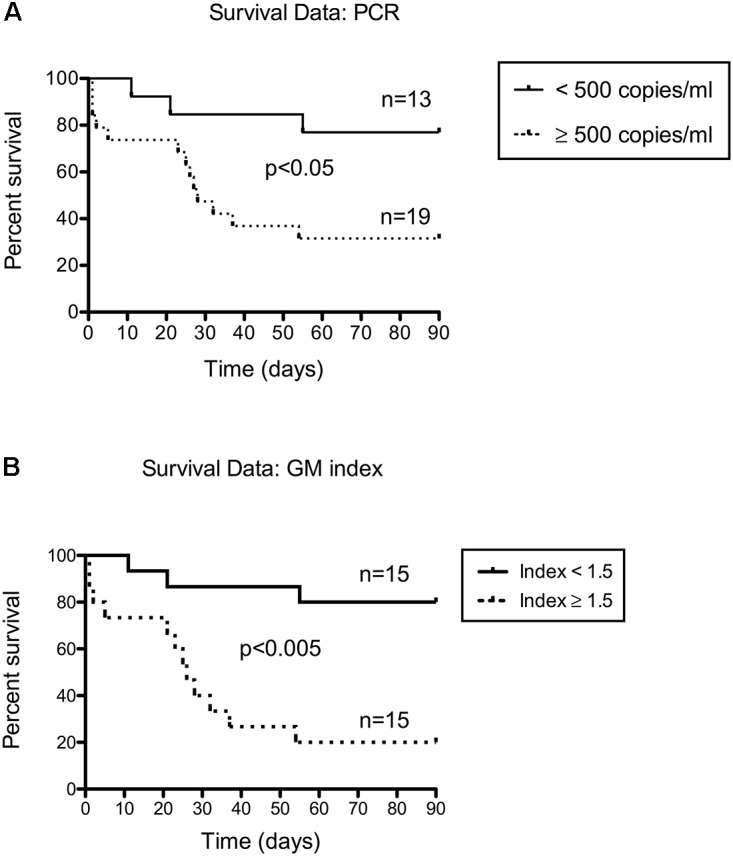
*Aspergillus* PCR performed in BAL is highly predictive of 90-day mortality in invasive pulmonary aspergillosis. **(A)** Patients with initial fungal loads below 500 copies/mL (*n* = 13; 76.9% survival) have more favorable outcomes than other patients (*n* = 19; 31.6% survival): *p* < 0.05 by Log-rank (Mantel-Cox) test; hazard ratio 0.23 (95% CI of ratio 0.08–0.65). **(B)** Patients with initial GM indices below 1.5 (*n* = 15; 80% survival) have more favorable outcomes than other patients (*n* = 15; 20% survival): *p* < 0.005 by Log-rank (Mantel-Cox) test; hazard ratio 0.16 (95% CI of ratio 0.05–0.47).

Furthermore, fungal load in BAL as a predictor of 90-day mortality in invasive pulmonary aspergillosis was analyzed. For this analysis, three PCR-negative invasive cases (one due to *A. nidulans* [not detected by the employed PCR technique] and two with no lung involvement) were excluded. ROC curves indicated that a cut-off of 500 copies/mL was the optimal threshold (**Supplementary Figure S1**). Patients with PCR results strictly below 500 copies/mL had a significantly higher probability of survival 90 days after the diagnosis (*n* = 10/13; 76.9% survival), compared to those with PCR results at or above this cut-off (*n* = 6/19; 31.6% survival; median survival of 28 days) (*p* < 0.05 with Log-rank test) (**Figure 4A**). The test had a hazard ratio of 0.23 (95% CI of ratio: 0.08–0.65). The characteristics of the patients (i.e., age, sex ratio, underlying diseases or antifungal therapy) as well as the interval between sampling and the start of targeted antifungal therapy were not different between these two groups (**Table 1**). Considering all invasive cases in our study due to *A. fumigatus* (even non-pulmonary forms) we found a less statistically significant result with a *p* = 0.05 (data not shown). Interestingly, the GM index in BAL supernatant also permitted this kind of discrimination with a 1.5 index cut-off, provided that the numerous false-negative tests were included in the analysis (**Figure 4B**). Patients with GM indices below 1.5 had a significantly higher probability of survival 90 days after the diagnosis (*n* = 12/15; 80% survival), compared to those with results above this cut-off (*n* = 3/15; 20% survival; median survival 26 days) (*p* < 0.005 with Log-rank test). The test had a hazard ratio of 0.16 (95% CI of ratio: 0.05–0.47).

## Discussion

*Aspergillus* DNA detection for the diagnosis of aspergillosis has been the subject of studies for more than 20 years (Yamakami et al., 1996). Numerous authors, having demonstrated sensitivities ranging from 72 to 88% and specificities from 75 to 98.7%, are now stating that *Aspergillus* PCR in blood is an interesting tool for the diagnosis of invasive aspergillosis in at-risk patients (Mengoli et al., 2009; Arvanitis et al., 2014, 2015; Imbert et al., 2016). Nonetheless, for the moment, expert consensus recommends its use only with certain precautions and opinions still diverge (Patterson et al., 2016). Furthermore, we note that in the literature, the performance of *Aspergillus* PCR has been assessed in blood samples much more frequently than it has in respiratory samples, such as BAL or sputum. Above all, the literature is essentially focused on patients with hematological conditions (Sun et al., 2011; Torelli et al., 2011; Hoenigl et al., 2014; Boch et al., 2016), and few included other populations (Luong et al., 2011; Guegan et al., 2018).

Our present work indicates that PCR performed in BAL is of particular interest for the diagnosis of invasive aspergillosis, providing 88.6% sensitivity and 95.5% specificity. These values are similar to those reported in previous reports, which showed sensibilities ranging from 71 to 82% and specificities from 92.8 to 98% (Tuon, 2007; Luong et al., 2011; Grancini et al., 2018; Guegan et al., 2018). Particularly, the rate for sensitivity observed for PCR in BAL was superior to those observed for the GM index whatever the cut-off used (0.5 or 1), and mycological culture. This was already demonstrated for mycological culture, but it is in contradiction with previous report for GM, which showed higher sensitivity than PCR (Guegan et al., 2018). We also underline that sensitivity was 96.9% when the analysis was limited to cases of invasive aspergillosis with pulmonary involvement and *A. fumigatus* as the causative species. The last pulmonary case was diagnosed with clinical and radiological evidences and positive GM and PCR in serum. Culturing is always performed as it enables the isolation of any fungus, i.e., yeast and mold. Retrieving the strain also permits the evaluation of the susceptibility of the fungus to antifungal drugs via the determination of minimal inhibitory concentrations. GM determination is strongly recommended for diagnosis and screening in patients with hematological conditions but not those with other conditions, such as solid organ transplant recipients (Patterson et al., 2016). In contrast, our work indicates that PCR is interesting in both hematological and non-hematological patients, including those solid organ transplant recipients who account for a third of the concerned population. These, demonstrate its usefulness in intensive care unit patients, whom diagnosis of IA remains difficult and needs improvements, as previously reported (Guegan et al., 2018).

The high sensitivity of PCR is also useful for the diagnosis of chronic aspergillosis forms, and its NPV is interesting whatever the form. The positivity of PCR in respiratory samples may be due to invasive aspergillosis, chronic aspergillosis or a clinically irrelevant situation (i.e., contamination or fortuitous colonization). Interestingly, we found that fungal load can distinguish between aspergillosis and non- aspergillosis pathologies and thus may be useful for discriminating between infection and colonization. However, in our study and in contradiction with a previous work (Luong et al., 2011; Grancini et al., 2018), fungal load was not able to distinguish between invasive and non-invasive forms. This finding was not particularly surprising as both invasive and chronic pathologies may involve a high fungal burden.

Although of particular interest for the diagnosis of aspergillosis, bronchoalveolar lavage is an endoscopic procedure and thus obtaining samples may be difficult in more fragile patients. We note that the proportions of patients with hematological conditions and of patients who were neutropenic were smaller in this study than in our previous work on PCR in serum. In that work, we also reported that the initial fungal load determined by PCR in serum was highly predictive of 90-day mortality (Imbert et al., 2016). Therefore, although BAL is not a physiological sample and is subject to procedure variations affecting its dilution, we investigated whether *Aspergillus* DNA quantification in BAL could be used to predict the outcome of invasive disease. When we focused on invasive pulmonary aspergillosis, we found that a PCR threshold of 500 copies/mL could discriminate patients with low (below the threshold) or high (above) probabilities of 90-day mortality. Of note, the GM index also discriminates between these two groups, but for this analysis, it included numerous false negatives (i.e., with a cut-off < 1). This suggests that GM may be better suited for the prediction of outcome rather than diagnosis.

There are nonetheless several limitations in our study. First, there is no reliable gold standard for the diagnosis of IA, and the majority of our cases are probable IAs. Evaluate diagnosis performances of the tests, may so include some bias. To avoid inclusion bias for the tests we wanted to evaluate (i.e., GM and PCR in BAL), we excluded them from the diagnosis criteria. Secondly, the PCR we use is specific to the *A. fumigatus* species and thus cannot amplify the genomes of other important species such as *A. flavus*, or *A. niger*. However, looking at the cases of invasive aspergillosis with a positive culture—and therefore an identification at the species level—there was only one case of non-*fumigatus* (*A. nidulans*) diagnosed in our series, which echoes findings from one of our previous studies (Imbert et al., 2016). Furthermore, even commercial multiplex approaches can lack relevant species such as *A. nidulans* (Chong et al., 2016). The last limitation is that this method is not designed to detect antifungal drug resistance, particularly Cyp51A-mediated azole resistance. However, that involves treatment rather than diagnosis. This latter remains the main purpose of *Aspergillus* PCR, but the technique could be improved by adding a Cyp51A gene-targeted PCR (Buil et al., 2018).

## Conclusion

*Aspergillus* DNA detection in bronchoalveolar lavage fluid by PCR is a very interesting tool for the diagnosis of invasive aspergillosis as its sensitivity is far better than those of the GM index and mycological examination. PCR performed in BAL is also of particular interest for the diagnosis of non-invasive aspergillosis. It also offers a high negative predictive value for all forms of aspergillosis. However, the PCR method employed in this study was not designed to detect non-fumigatus species; efforts are necessary to overcome that limit. Moreover, PCR permits the quantification of fungal load, which, in positive cases, might be used to discriminate between aspergillosis and contamination or clinically irrelevant colonization. The results of this study also indicate that in cases of invasive pulmonary aspergillosis, a threshold of 500 copies/mL is useful to discriminate patients with low (below the threshold) or high (above) probabilities of 90-day mortality. This cut-off might be used on one hand to identify patients needing particularly intensive care and on the other to design further clinical studies.

## Author Contributions

SI performed the data analysis and participated in the writing of the manuscript. IM, MP, J-YB, FT, and FG performed some experiments. MU and VT-D participated in scientific discussion and participated in the writing of the manuscript. AF designed the study, performed data analysis, and wrote the paper. All the authors contributed to manuscript revision, read and approved the submitted version.

## Conflict of Interest Statement

The authors declare that the research was conducted in the absence of any commercial or financial relationships that could be construed as a potential conflict of interest.

## Footnotes

1 http://marne.u707.jussieu.fr/biostatgv/
