# Supplementary material for: Aspergillus PCR in Bronchoalveolar Lavage Fluid for the Diagnosis and Prognosis of Aspergillosis in Patients With Hematological and Non-hematological Conditions
Source: Front Microbiol. 2018 Aug 14;9:1877. doi: 10.3389/fmicb.2018.01877 (PMC6102318; doi:10.3389/fmicb.2018.01877)

Figure S1: ROC curves investigating the efficiency of GM (left) and PCR (right) in BAL to predict the outcome (death) at day 90, in patients suffering from invasive pulmonary aspergillosis due to *A. fumigatus*


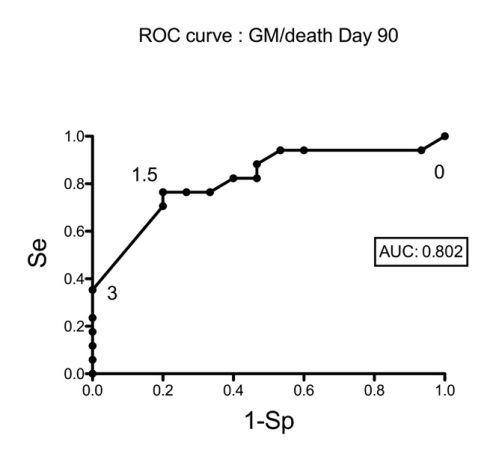

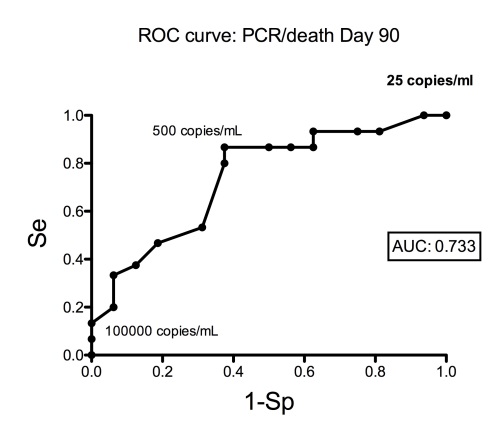

Supplement: Supplementary file 1 [file Data_Sheet_1.docx]
